# Overall survival and associated factors among patients with pulmonary Kaposi’s sarcoma in sub-Saharan Africa

**DOI:** 10.1371/journal.pgph.0006741

**Published:** 2026-06-26

**Authors:** Salum J. Lidenge, Herriethsiah S. Noah, Ernesti M. Zakayo, Felister M. Tupa, Emmanuel L. Lugina, John R. Ngowi, Saida K. Abeid, Revelian Iramu, Owen Ngalamika, Chacha J. Mwita, Charles Wood, Julius Mwaiselage

**Affiliations:** 1 Clinical Research Training and Consultancy Unit, Ocean Road Cancer Institute, Dar es Salaam, Tanzania; 2 Department of Clinical Oncology, Muhimbili University of Health and Allied Sciences, Dar es Salaam, Tanzania; 3 School of Public Health, Louisiana State University Health Sciences Center, New Orleans, Louisiana, United States of America; 4 Dermatology and Venereology Section, Adult Hospital of the University Teaching Hospitals, University of Zambia School of Medicine, Lusaka, Zambia; 5 Department of Interdisciplinary Oncology, Louisiana State University Health Sciences Center, New Orleans, Louisiana, United States of America; PLOS: Public Library of Science, UNITED STATES OF AMERICA

## Abstract

Visceral Kaposi’s sarcoma (KS), like pulmonary KS (PKS), is more common in HIV associated/epidemic KS (EpKS). Its presentation often mimics other opportunistic pulmonary infections and is associated with poor outcomes. This study investigated the prevalence of PKS, and factors associated with overall survival at a tertiary hospital in Tanzania. This retrospective study reviewed records of 269 histologically confirmed cutaneous KS patients treated at the Ocean Road Cancer Institute between January 2019 and December 2022. Sociodemographic, clinical, and survival data were extracted from patients’ files. Specialist radiologists reviewed chest radiographs taken at the time of diagnosis, categorizing them as normal, infectious infiltrates, or PKS. Data were analyzed using descriptive statistics, and Cox Proportional Hazard model identified factors linked to overall survival. Statistical significance was defined as p < 0.05. Among 269 patients with cutaneous KS, 195 had EpKS, 66 had Endemic KS (EnKS), and 8 had unknown HIV status and were excluded from further analysis. The male-to-female ratio was 2:1. While all patients had cutaneous KS lesions, 23 (8.8%) had PKS which was significantly more common in the EpKS than EnKS group (p = 0.024). Additionally, 24 patients (9.2%) had Chest X-ray findings (CXR) indicative of infection. At one year, overall survival was 53% for patients with PKS, compared with 87% for those without PKS. In the adjusted analysis, patients without PKS had an 86% lower risk of mortality than those with PKS (aHR = 0.14; 95% CI: 0.07–0.32; P < 0.001). There is high prevalence of advanced KS presentation and poor overall survival especially among PKS in SSA, despite widespread ART use. Although CXR remains the diagnostic mainstay in this setting, it is subject to notable limitations. Simple, low-cost diagnostic algorithms are needed to optimize CXR utility alongside the ongoing expansion of advanced imaging and bronchoscopic services in the region.

## Introduction

Kaposi sarcoma (KS) is an angioproliferative tumor that presents in one of four forms: classic KS (CKS), Endemic KS (EnKS), AIDS-related/Epidemic KS (EpKS), or iatrogenic KS [1]. The two most common forms in Africa are EnKS, also known as ‘African KS,’ which occurs in HIV-negative individuals primarily in sub-Saharan Africa (SSA), and EpKS, which occurs in HIV immunocompromised individuals [1–4]. Unlike EnKS, EpKS is an aggressive disease associated with visceral involvement and poor treatment response. All forms of KS are caused by Kaposi’s sarcoma-associated Herpesvirus (KSHV) [5].

In the West, the incidence of KS has declined since the introduction of combined Anti-Retroviral Therapy (cART) [6]. A similar decline in KS incidence has not been replicated in SSA despite widespread cART use [6]. Perhaps, factors like delayed HIV diagnosis, delay in initiation of cART, widespread KSHV infection, and delayed KS diagnosis contribute to the high KS burden [4,7,8]. Furthermore, there are reports of incident KS following controlled HIV disease (HIV viral load suppression and improved CD4 count) among people living with HIV (PLWH) on cART [8,9].

Pulmonary Kaposi’s sarcoma (PKS) is a serious manifestation of KS that is associated with advanced disease and generally poor outcomes [10–12]. In addition to prompt initiation and optimization of cART, better treatment outcomes of PKS have been reported by using liposomal anthracyclines and paclitaxel compared to conventional chemotherapy using Adriamycin, Bleomycin, and Vincristine (ABV) [13,14]. However, access to the first-line liposomal anthracyclines and paclitaxel is limited in SSA, and ABV remains the main chemotherapy regimen utilized for advanced KS [15,16]. Palliative radiotherapy can be used in instances of airway obstruction or pain control [17,18]. In refractory effusions, pleural effusion tapping can be used to relieve life-threatening symptoms and improve quality of life. Other treatment options include immunomodulatory agents like pomalidomide and immune checkpoint inhibitors like pembrolizumab for refractory patients [19,20].

Since the majority of the PKS cases occur in HIV immunocompromised individuals, sometimes it is challenging to differentiate PKS from opportunistic pulmonary infections like lymphoid interstitial pneumonia, Tuberculosis (TB), and Pneumocystis jirovecii pneumonia (PJP) [10,12,21,22]. Clinically, PKS can also mimic other KSHV-related pathologies, such as KSHV-associated primary effusion lymphoma and KSHV-associated multicentric Castleman’s disease unless specific markers like c-reactive protein, interleukin-6, and interleukin-10 are used to distinguish the conditions [23,24]. In the setting of Kaposi's sarcoma-associated immune reconstitution inflammatory syndrome (KS-IRIS), PKS can progress rapidly with increased risk of mortality [25].

Given this overlap, accurate and timely diagnosis often requires more advanced and multimodality investigations [11,26,27]. Otherwise, distinguishing PKS from other pulmonary pathologies can be challenging, potentially leading to delays in appropriate treatment and poor patients’ outcomes [26,27]. In SSA, significant diagnostic gaps persist in the availability and access to standardized PKS diagnostic investigations. Clinical diagnosis supplemented by chest X-ray (CXR) imaging remains the primary imaging modality for PKS diagnosis in most instances. Prior studies have shown that characteristic CXR features, combined with clinical symptoms, can provide a reasonably acceptable PKS diagnosis in the absence of advanced imaging with High-Resolution Computed Tomography (HR-CT) and histopathological confirmation in resource-limited settings [28,29]. Therefore, this study used clinical respiratory symptoms and CXR findings to diagnose PKS and assess the prevalence of PKS and factors associated with overall survival among patients with cutaneous KS at the Ocean Road Cancer Institute (ORCI), Dar es Salaam, Tanzania.

## Materials and methods

### Study design and subjects

This was a retrospective hospital-based study that reviewed records of all patients with cutaneous KS who attended the ORCI between January 2019 and December 2022. ORCI is the main national public cancer referral center in Tanzania, receiving cancer patients from all over the country and neighboring countries in Eastern, Southern, and Central Africa. Records of 269 histologically confirmed patients with KS were included in the study. Sociodemographic, clinical, and survival information relevant to the study were extracted using a standardized data collection form.

Routine chest radiographic images obtained at the time of KS diagnosis were reviewed and reinterpreted by two independent specialist radiologists. Following the review, CXR findings were classified as normal chest findings, infectious infiltrates, or pulmonary Kaposi’s sarcoma (PKS). Concordant PKS interpretations between the two specialist radiologists were considered definitive. Normal CXR findings and infectious infiltrates CXR findings were further categorized as non-PKS. All CXR imaging were performed using a SITEC DigiRAD-FP digital X-ray unit. Because CXR was routinely conducted for all patients at diagnosis, no records were excluded from the study based on the imaging availability.

Time-to-event (death) information was collected retrospectively from patient medical records, although most deaths occurred at home. The outcome of interest was overall survival, defined as the time from the date of first presentation at ORCI following histological confirmation of KS to one year after treatment completion, measured in months. Patients who were alive at their last recorded clinic visit, lost to follow-up, or transferred to another facility were censored at their last contact date. Loss to follow-up was defined as the absence of clinical follow-up for six or more consecutive months.

Other covariates included sociodemographic characteristics (age, sex, geographical location), lesion characteristics (distribution and morphotype), cART use, and treatment modality. The cART use was defined as documented enrollment in HIV care and at least three months of therapy. Treatment modalities were classified into watchful waiting, chemotherapy, radiotherapy, or palliative/supportive care based on clinical presentation. Chemotherapy regimens were grouped as paclitaxel or Adriamycin, bleomycin, and vincristine (ABV) combination.

Ethical approval for this study was obtained from the Tanzania National health research ethics committee (NaTHREC - NIMR/HQ/R.8a/Vol. IX/3750). At initial registration at ORCI, all patients provided written informed consent for treatment and the use their anonymized clinical data for research purposes. For patients under 18 years of age, written informed consent was provided by a parent or legal guardian at the time of care. All data were fully de-identified prior to analysis. Unique file numbers were retained for data linkage by researchers at ORCI, while all personal identifiers, including names, were removed to ensure confidentiality. As this was a retrospective study using secondary data, the requirement for additional informed consent was waived by the ethics committee.

### PKS definition

PKS was diagnosed based on a combination of histologically confirmed cutaneous KS, the presence of clinical respiratory symptoms, and characteristic CXR findings. Respiratory symptoms included non-productive cough, shortness of breath, coughing up blood, and chest pain. In contrast, the radiological features included flame signs, a metastatic pleural effusion, mediastinal lymphadenopathy, and reticulonodular opacities with prominence in the fissures, the perihilar mid to lower lung regions, and peribronchovascular areas. It should be noted that the use of CXR findings for PKS diagnosis is a pragmatic approach rather than a gold-standard imaging modality. The high-resolution CT scan (HR-CT), a standard diagnostic imaging modality for PKS, is not commonly used in SSA due to limited affordability. Therefore, CXR remains the most common modality in the region.

### Statistical analysis

All data abstraction forms were checked for completeness before entry into an MS Excel database. Data analysis was performed using R (version 4.x.x) within the RStudio integrated development environment (RStudio version 2023.x). The Shapiro–Wilk test was used to assess the normality of continuous variables. Quantitative variables that were normally distributed were summarized as mean ± standard deviation (SD), while non-normally distributed variables were summarized as median and interquartile range (IQR). Categorical variables were summarized using frequencies and percentages. Comparisons of means were performed using the Student’s t-test, and comparisons of medians were conducted using the Mann–Whitney U test. Survival probabilities were estimated using the Kaplan–Meier method, and the prognostic significance of variables was evaluated using the log-rank test in univariate analysis. Variables with p-values < 0.2 in the univariate analysis were further analyzed in multivariable Cox proportional hazards regression models. Variables entered into the Cox regression model included age, presence of pulmonary Kaposi’s sarcoma (PKS), histology/morphotype, lesion distribution, and gender. Hazard ratios (HRs) with 95% confidence intervals (CIs) were reported. The proportional hazards assumption was evaluated using Schoenfeld residuals (cox.zph function in R). Both individual covariates and the global test were examined. A p-value > 0.05 was considered evidence that the proportional hazards assumption was not violated. Additionally, scaled Schoenfeld residual plots for each covariate were visually inspected for deviations from linearity. A sensitivity analysis was conducted among records with available CD4 counts to explore the effect of PKS on 1-year overall survival. A p-value of less than 0.05 was considered statistically significant.

## Results

### Sociodemographic and clinical characteristics of the study subjects

In this study, 269 records of patients with cutaneous KS were retrieved. The final analysis included 261 records, of which 195 (74.7%) had epidemic KS (EpKS), and 66 (25.3%) had endemic KS (EnKS). Eight patients (3%) had no documented HIV serostatus and were therefore excluded from further analysis. Most patients 157 (60.2%) were from the coastal zone of Tanzania, and 4 (1.4%) patients were from neighboring countries, including Malawi (2 patients), Zambia (1 patient), and the Comoro Islands (1 patient).

Although high-resolution computed tomography (HR-CT) is the standard imaging modality for diagnosing PKS, only nine patients (3.3%) underwent chest CT for evaluation. The remaining patients were assessed using a CXR. A total of 23 patients (8.8%) had pulmonary Kaposi sarcoma (PKS), while 238 (91.2%) did not. Among participants without PKS, most had normal chest X-ray (CXR) findings 214(89.9%), while 24(10.1%) had radiographic features suggestive of infectious processes. Other than a few confirmed cases of pulmonary TB and infection of the KS lesions, other co-infections were largely not documented.

The overall median age at presentation was 43.7 years (IQR: 34–52). Patients with PKS were significantly younger compared to those without PKS (median age: 37 vs. 43 years, p < 0.001). Most of the patients were males with a male-to-female ratio of about 2:1 in the entire cohort, however, this usual male predominance was lost among patients with PKS where male-to-female ratio was 1:1. Generally, sex distribution did not differ significantly by PKS status (p = 0.134). Similarly, marital status distribution was comparable among both outcome groups and employment status was not significantly associated with PKS. About 92.3% of patients with KS in this study (n = 241) were recorded as unemployed.

Regarding clinical characteristics, 26.1% of patients had disseminated cutaneous disease. This presentation was more common among patients with PKS 14 (60.9%) than among patients without PKS 54 (22.7%) (p < 0.001). Additionally, approximately one-third of the records with the histologically examined lesions were nodular cutaneous lesions, which is considered an advanced presentation, however, lesion morphotype did not differ significantly between those with and without PKS (p = 0.684).

Lastly, HIV seropositivity was significantly more common among patients with PKS compared to those without PKS (95.7% vs 72.7%, p = 0.016). Of the 261 patients’ records, only 17 (6.5%) had recorded CD4 counts, with 8 measured at baseline and 9 obtained later after initiating cART. The median CD4 count was 316 cells/µL (interquartile range, 223–515). Among these 17 patients’ records, 1 had PKS, and 16 did not. A sensitivity analysis was conducted among patients with available CD4 measurements to examine 1-year overall survival by PKS status. Cox proportional hazards regression was attempted to adjust for CD4 count; however, due to the extremely small sample size and only 1 observed PKS case in this subset, the estimates were highly unstable. Majority of the patients 188 (96%) reported use of cART of at least 3 months. However, cART use did not differ significantly across outcome groups (p = 0.337). The most common cART regimen used was Tenofovir, Lamivudine and DTG combination (Table 1).

**Table 1:** Sociodemographic, clinicopathological features, and treatment modalities of the study participants (N = 261).

Variable		Pulmonary Kaposi Sarcoma (n, %)		Total (N = 261)	P-value
	Levels	With PKS 23(8.8)	Without PKS 238(91.2)		
**Age (years)**	**Median Age (IQR)**	37(29-45)	43(34-53)	43(34-52)	<0.001
**Age (years)**	**13-35**	10(43.5)	70(29.4)	80 (30.7)	0.089
	**36-50**	11(47.8)	98(41.2)	109(41.8)	
	**>50**	2(8.7)	70(29.4)	72(27.6)	
**Sex**	**Male**	12 (52.2)	161 (67.6)	173 (66.3)	0.134
	**Female**	11(47.8)	77 (32.4)	88(33.7)	
**Marital status**	**Married**	9 (39.1)	65 (27.3)	74(28.4)	0.703
	**Single**	14 (60.9)	173 (72.7)	187(71.6)	
**Occupation**	**Employed**	0	20 (8.4)	20 (7.7)	0.148
	**Not Employed**	23 (100.0)	218 (91.6)	241 (92.3)	
**Zones Distribution**	**Coast**	16 (69.7)	141 (59.2)	157 (60.2)	0.334
	**Others**	7 (30.4)	97 (40.8)	104 (39.8)	
**Lesions Distributions**	**Disseminated**	14 (60.9)	54 (22.7)	68(26.1)	<0.001
	**Non-Disseminated**	9 (39.1)	184 (77.3)	193 (73.9)	
**Lesions morphotype**	**Nodular stage**	8 (34.4)	73 (30.7)	81 (31.0)	0.684
	**Others**	15 (65.2)	165 (69.3)	180 (69.0)	
**HIV Status**	**Positive**	22 (95.7)	173 (72.7)	195 (74.7)	0.016
	**Negative**	1(4.3)	65 (27.3)	66 (25.3)	
**cART use**	**Yes**	22 (100.0)	166 (96.0)	188 (96.3)	0.337
	**Not Documented**	0	7 (4.0)	7(3.6)	
	**N/A**	1	65	66	
**Treatment Modality**	**Watchful Waiting**	0	15(6.3)	15(5.7)	<0.001
	**Chemotherapy**	21 (91.3)	81 (34.1)	102 (39.1)	
	**Radiotherapy**	1 (4.3)	136 (57.1)	137 (52.5)	
	**Palliative/ supportive care**	1 (14.3)	6 (2.5)	7 (2.7)	
**Chemotherapy Cycles (n = 102)**	**>3 cycles**	10 (47.6)	63 (77.8)	73 (71.6)	0.006
	**≤ 3 cycles**	11 (52.4)	18 (22.2)	29 (28.4)	
**Chemotherapy Regimen (n = 102)**	**Paclitaxel**	9 (42.9)	42 (51.9)	51 (50.0)	0.463
	**ABV**	12 (57.1)	39 (48.1)	51(50.0)	

»N/A – Not Applicable, ABV - Adriamycin, Bleomycin, and Vincristine

* Percentages (Yes/No) are calculated within each category of the outcome variable (column%)

### Treatment modalities employed during the study

In this study, the treatment modalities included radiotherapy, chemotherapy, watchful waiting, and palliative supportive management, all in conjunction with cART use for EpKs patients. Radiotherapy was the most common modality 137(52.5%) and it was prescribed for patients whose KS lesions were localized and limited to the extremities. The radiotherapy dose ranged between 6Gy and 8Gy per fraction, given as a single fraction to the affected area and repeated up to 3 times at an interval of 1 month per fraction, depending on disease response. Most of the patients without PKS were treated with radiotherapy, 136 (57.1%).

A total of 102 (39.1%) received either single-agent chemotherapy (Paclitaxel) or a combination of Adriamycin, Bleomycin, and vincristine (ABV), depending on their performance status at presentation and the affordability of chemotherapy. While ABV was readily available and affordable, paclitaxel use was restricted by patients’ affordability. ABV was administered on a 3-weekly schedule, while paclitaxel was administered on a bi-weekly schedule, following hematological profile assessment. Most patients diagnosed with PKS 21 (91.3%) received chemotherapy, of whom 12 (57.1%) received the ABV regimen. The number of treatment cycles completed among patients who received chemotherapy (n = 102), differed significantly across outcome groups (p = 0.006). About 47.6% of those with PKS received more than three cycles, while 77.8% of those without PKS received more than 3 cycles of chemotherapy.

Seven (2.7%) patients received supportive palliative care in the form of pain management and wound care due to poor performance status, and the other 15 (5.7%) patients were maintained on cART and active surveillance. The ones kept on active surveillance had discrete lesions and were followed up every 3 months. (Table 1).

### Overall survival among patients with KS in the study

A total of 30 (13.4%) patients were considered lost to follow-up at the end of the one-year follow-up period where 4 (11.4%) of those were patients with PKS.

**Fig 1:**
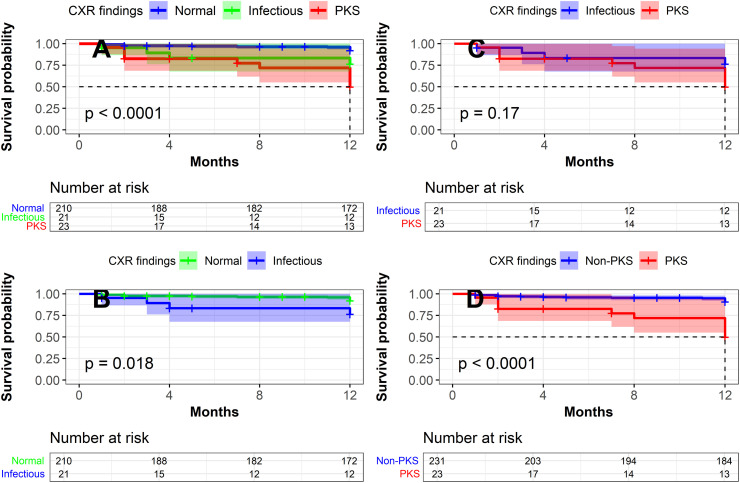
Kaplan Meir curves showing overall survival among patients with KS. **A.** Overall survival of patients with KS based on chest X-ray (CXR) findings, **B.** Subgroup analysis comparing 1-year OS of patients with KS with normal CXR findings versus CXR suggestive of infectious infiltrates, **C.** Subgroup analysis comparing 1-year OS of patients with KS with CXR suggestive of infectious infiltrates versus CXR suggestive of PKS, and **D.** Overall survival of patients with KS without PKS versus those with CXR suggestive of PKS. KS is Kaposi’s sarcoma, PKS is Pulmonary Kaposi’s sarcoma.

To assess overall survival, we performed Kaplan-Meier analysis with a log-rank test to compare overall survival (OS) by chest imaging findings. The 1-year OS was 90% for patients with normal CXR findings, 75% for those with infectious infiltrates, and 50% for those with CXR suggestive of PKS (p < 0.0001) (Fig 1A).

Subgroup analysis comparing 1-year OS of patients with KS with normal CXR findings versus CXR suggestive of infectious infiltrates revealed that 1-year OS for patients with normal CXR findings was significantly higher than for patients with CXR findings suggestive of infectious infiltrates (p = 0.018) (Fig 1B). However, there was no statistically significant difference between patients with CXR suggestive of infectious infiltrates and those with CXR suggestive of PKS (p = 0.17) (Fig 1C). This suggests that the use of CXR in PKS diagnosis likely underestimates the burden and misclassifies some PKS cases as infectious infiltrates. Overall, the median survival time for patients with PKS was 12 months, while the patients without PKS did not reach a median survival time. Patients with PKS had a lower 1-year OS (53%) than those with non-PKS CXR findings (87%) (p < 0.0001) (Fig 1D).

**Fig 2:**
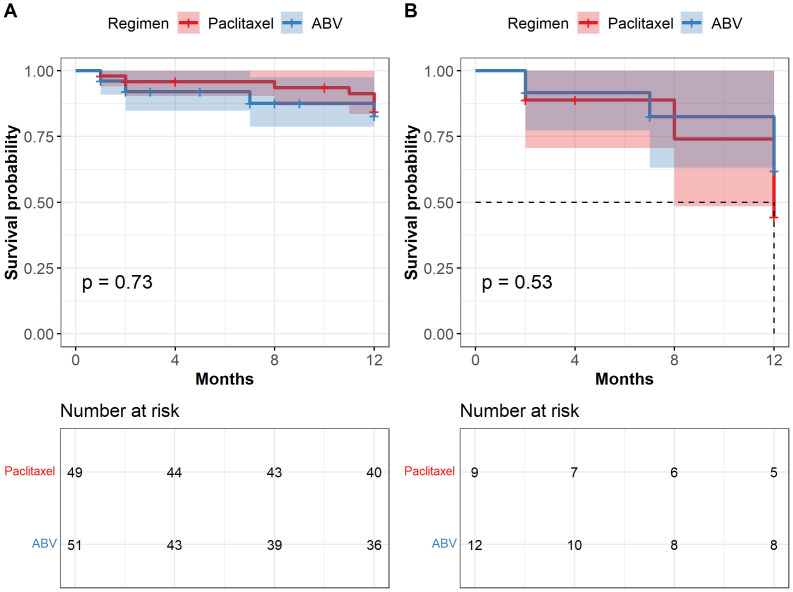
Kaplan Meir survival curves showing overall survival among patients with KS by chemotherapy regimen. **A.** Overall survival of all patients with KS who received chemotherapy, and **B.** Subgroup analysis comparing Paclitaxel and ABV regimen among patients with PKS**.** KS is Kaposi’s sarcoma. PKS - Pulmonary Kaposi’s sarcoma, ABV - Adriamycin, Bleomycin, and Vincristine.

We also compared overall survival among patients with KS by chemotherapy regimen. There was no statistical difference in 1-year OS between the Paclitaxel and ABV regimens in the entire cohort (Fig 2A). In a subgroup analysis comparing the Paclitaxel and ABV regimens among patients with PKS, there was also no statistical difference in 1-year OS between the regimens (Fig 2B).

To further assess factors influencing mortality, we conducted a Cox proportional hazards analysis. In univariate Cox proportional hazards analysis, chest imaging finding was significantly associated with overall survival, while other covariates including age, sex, geographic distribution, HIV status, lesion morphotype, and treatment modality were not statistically significant. Patients with PKS had a 6.37 times higher risk of death compared to those without PKS in the univariate analysis (cHR = 6.37; 95% CI: 2.95–13.73; P < 0.001). After adjusting for other prognostic factors in a multivariate Cox proportional hazards model, the risk of mortality remained significantly higher for patients with PKS (aHR = 7.15; 95% CI: 2.97–17.2; P < 0.001) (Table 2). The proportional hazards assumption was assessed using Schoenfeld residuals and was not violated (p = 0.42; see S1 Fig). Cohen’s D for PKS was 1.29, indicating a large effect size (S1 Table).

**Table 2:** Univariate and multivariate analysis of factors associated with death among patients with KS.

Variable		Univariate analysis				Multivariate analysis		
		p-value	cHR	95% CI	p-value		aHR	95% CI
**Age**		0.262	0.98	0.96-1.01	0.63		0.99	0.96-1.02
**Gender**	**Male**	0.791	1.11	0.51-2.44	–		–	–
	**Female**	1			–		–	–
**HIV status**	**Positive**	0.321	1.63	0.61-4.27	–		–	–
	**Negative**	1			–		–	–
**Cutaneous Lesion morphotype**	**Nodular**	0.291	0.17	0.22-1.35	0.08		0.45	0.18-1.13
	**Non-nodular**	1			–		–	–
**KS Lesions distribution**	**Disseminated**	0.169	0.59	0.28-1.25	0.86		0.92	0.39-2.18
	**Non-disseminated**	1			1		–	–
**Chemotherapy regimen**	**Paclitaxel**	0.736	1.19	0.43-3.29	–		–	–
	**ABV**	1			–		–	–
**Number of chemotherapy cycles**	**>3**	0.256	1.9	1.1-3	–		–	–
	**≤3**	1			–		–	–
**PKS**	**Yes**	<0.001	6.37	2.95-13.73	<0.001		7.15	2.97-17.2
	**No**	1			1		–	–

» cHR-Crude Hazard Ratio, PKS-Pulmonary KS, aHR-Adjusted Hazard Ratio, ABV -Adriamycin, Bleomycin, and Vincristine.

## Discussion

In sub-Saharan Africa (SSA), KS is a widespread malignancy and remains a significant health concern, particularly among people living with HIV (PLWH) [22,30]. Cutaneous presentation is common; however, visceral involvement can occur and is often regarded as a disease progression that predicts poor treatment outcome [9,31]. During the pre-cART era, autopsy findings revealed that visceral involvement, particularly PKS, was common, affecting approximately 45% of patients with EpKS who already had diagnosed cutaneous KS lesions [28]. In this study, we investigated the burden of visceral KS at presentation, specifically PKS, and factors associated with the overall survival among 261 patients diagnosed with KS at a major cancer referral hospital in SSA. The prevalence of PKS among individuals diagnosed with KS in this study was 8.8% and was primarily observed among those with epidemic KS. Similarly high prevalence of PKS was reported in the early years of cART by Palmieri et al. using the Chelsea and Westminster Cohort in Europe in 2006 [10]. Now that cART is well-established and known to be effective, the prevalence of PKS was expected to be low. However, PKS in the cART era remains high not only in SSA [32]. Several factors are implicated including the high levels of immunosuppression before cART initiation, as indicated by low CD4 counts in some cases, non-compliance, or KS-Immune Reconstitution Syndrome (KS-IRIS) that occurs upon initiation of cART [10,28,31]. In this study, although most patients were on cART for at least 3 months, we were unable to assess immune status (CD4 count and viral load) or cART adherence due to missing information in the secondary data. Proper documentation of clinical records, including the establishment of standardized minimum datasets that contain essential data elements, and cause of death is recommended to support future studies on the treatment outcomes of PLWH.

Access to chest CT scan remains limited in much of SSA, largely due to financial constraints [22,31,33]. In our study, HR-CT scan, the recommended diagnostic modality, was performed in only 9 (3.3%) patients due to affordability. Consequently, most patients underwent CXR imaging for pulmonary evaluation. Importantly, none of the PKS diagnoses were made using bronchoscopy and biopsy for histological confirmation. Although respiratory signs and symptoms and CXR imaging for PKS diagnosis is acceptable in the region, it should be emphasized that the lack of HR-CT and bronchoscopy might have led to misclassification and underestimation of the PKS burden. For instance, 10% of patients were conservatively diagnosed with infectious conditions, although it is known that in the context of HIV co-infection, CXR imaging findings can be challenging to distinguish from PKS [31,34]. The fact that the OS of patients with CXR findings suggestive of infectious infiltrates was not significantly different from that of patients with PKS supports the possibility of misclassification of some PKS lesions, leading to an underestimation of the disease burden in the region. Despite the noted diagnostic limitations in this study, CXR imaging identified patients with PKS, and CXR findings were a significant predictor of death. Future studies on low-cost diagnostic algorithms that enhance the yield of CXR imaging are needed in SSA. The use of artificial intelligence (AI) to enhance the diagnostic accuracy of CXR imaging can be an alternative. For example, in lung cancer, AI algorithms combined with chest radiographs have improved detection and reduced the number of unnecessary CT scans to patients [35]. Similar studies are needed in the future to investigate the feasibility of using AI algorithms on PKS diagnosis in low-resource settings including the potential use of training datasets specific to African populations. Improving radiology capacity in the region, including enhancing access to tomography and bronchoscopy for PKS confirmation, is also needed.

Current treatment options for advanced KS include liposomal anthracyclines, paclitaxel, pomalidomide, and immune checkpoint inhibitors such as pembrolizumab [15,36–38]. In SSA, the cost of these drugs is prohibitive for most patients; therefore, conventional chemotherapy in the form of ABV remains the main option [15,16]. In our study, most of the patients presented with advanced disease, and ABV was frequently used. Although CXR findings were significantly associated with poor overall survival, the use of less effective ABV could have also contributed. It must be noted that other potential variables, such as immune status, HIV viral load, cART adherence, and comorbidities, that could have influenced overall survival were not investigated in this study. Surprisingly, the reported superiority of paclitaxel over ABV in overall survival was not observed in this study [13,14]. This lack of difference could also be due to the small sample size, particularly among patients with PKS and advanced disease at presentation.

### Implications for clinical practice in sub-Saharan Africa

Based on our findings, several practical considerations may improve the care of individuals with PKS in sub-Saharan Africa. Early recognition of pulmonary involvement among people with KS particularly in PLWH, should be prioritized through routine assessment of respiratory symptoms and timely use of chest radiography. Given limited access to advanced imaging, strengthening the diagnostic utility of chest X-ray through standardized interpretation protocols and training may enhance early detection. Integration of HIV and oncology care is essential to ensure timely initiation and adherence to cART and chemotherapy. In addition, improving clinical documentation, including immune status, treatment adherence, and causes of death is critical for both patient management and research. Where feasible, expanding access to diagnostic tools such as CT scanning and bronchoscopy, alongside investment in low-cost innovations such as AI–assisted imaging, may further improve diagnostic accuracy. Finally, strategies to reduce loss to follow-up, including patient tracking and decentralized care models, are needed to improve survival outcomes.

### Study limitations

During this study, several limitations were noted; the missing data on HIV status, CD4 counts, and HIV viral load was crucial since immune status is central to PKS pathogenesis. This has limited our ability to correlate patients’ immune status and the risk of PKS diagnosis and overall survival. While the study was conducted at a major cancer referral center, which meant the patients represented the KS population in the country, there was potential referral/selection bias. Most patients referred to the tertiary center tend to have advanced disease compared to those in primary and secondary centers, and that might have influenced the high PKS prevalence and poor overall survival. In addition, this study included only histologically confirmed cases of KS. Data on individuals with clinically suspected KS who did not undergo biopsy were not available. As a result, individuals with more severe disease who may have died before diagnostic confirmation could have been systematically excluded, potentially introducing survival bias leading to an underestimation of disease severity and mortality. Furthermore, about 11% of patients with KS in this study were lost to follow-up. Patients lost to care often experience severe debilitation or death, and such occurrences result in incomplete data. This attrition skews survival data, making it difficult to generalize conclusions. The low number of patients with PKS in this study also limited the generalizability of some of our findings. Therefore, larger prospective studies that integrate HIV and KS treatment services to improve care retention are needed to validate some of this study's findings.

## Conclusion

As observed in this study conducted at a tertiary cancer referral center, the prevalence of PKS is still high in SSA despite widespread cART. Advanced disease presentation is common, and the overall survival of patients with KS is low, particularly among those with PKS. Albeit with some caveats, CXR imaging remains the most common PKS diagnostic tool in SSA. Therefore, simple, low-cost diagnostic algorithms that enhance CXR image quality and interpretation are needed, while access to tomography and bronchoscopy is expanding in the region.

## Supporting information

S1 Fig Proportional Hazards (PH) assessment plot for each covariate in the Cox proportional hazards model. Based on these visual assessments for each covariate, supported by the non-significant p-values (all p > 0.05) in the Schoenfeld residuals test, there is no strong evidence to suggest a violation of the proportional hazard assumption for any of the covariates in the model. This indicates that the Cox proportional hazards model is a good fit for the data, as the effect of each predictor on the hazard appears to remain constant over time.
(TIF)

S2 Fig Representative CXR images retrieved from records of patients with KS. A) A representative image showing bilateral asymmetrical ill-defined reticulonodular opacities more on the middle and lower lobes of the lungs, while B) a representative image showing bilateral poorly defined infiltrates more on the lower zones involving the peribronchial region, with mild right-sided pleural effusion. All these images were classified as PKS.
(TIF)

S1 Table A summary of the Schoenfeld residual test for the proportional hazard assumption » PKS-Pulmonary KS, df-degree of freedom. (DOCX)

## References

[1] Cesarman E, Damania B, Krown SE, Martin J, Bower M, Whitby D. Kaposi sarcoma. Nat Rev Dis Primers. 2019;5(1):9. doi: 10.1038/s41572-019-0060-9 30705286 PMC6685213

[2] Fu L, Tian T, Wang B, Lu Z, Gao Y, Sun Y, et al. Global patterns and trends in Kaposi sarcoma incidence: a population-based study. Lancet Glob Health. 2023;11(10):e1566–75. doi: 10.1016/S2214-109X(23)00349-2 37734800

[3] Zeinaty PE, Lebbé C, Delyon J. Endemic Kaposi’s Sarcoma. Cancers (Basel). 2023;15(3). 10.3390/cancers15030872 PMC9913747 36765830

[4] Motlhale M, Sitas F, Bradshaw D, Chen WC, Singini MG, de Villiers CB, et al. Epidemiology of Kaposi’s sarcoma in sub-Saharan Africa. Cancer Epidemiol. 2022;78:102167. doi: 10.1016/j.canep.2022.102167 35504064

[5] Lidenge SJ, Tso FY, Ngalamika O, Ngowi JR, Mortazavi Y, Kwon EH. Similar immunological profiles between African endemic and human immunodeficiency virus type 1–associated epidemic Kaposi sarcoma (KS) patients reveal the primary role of KS-associated herpesvirus in KS pathogenesis. J Infect Dis. 2019;219(8):1318–28. doi: 10.1093/infdis/jiy654 30452681 PMC6452303

[6] Semeere AS, Busakhala N, Martin JN. Impact of antiretroviral therapy on the incidence of Kaposi’s sarcoma in resource-rich and resource-limited settings. Curr Opin Oncol. 2012;24(5):522–30. doi: 10.1097/CCO.0b013e328355e14b 22729153 PMC3418488

[7] Liu Z, Fang Q, Zuo J, Minhas V, Wood C, Zhang T. The world-wide incidence of Kaposi’s sarcoma in the HIV/AIDS era. HIV Med. 2018;19(5):355–64. doi: 10.1111/hiv.12584 29368388

[8] Rohner E, Valeri F, Maskew M, Prozesky H, Rabie H, Garone D, et al. Incidence rate of Kaposi sarcoma in HIV-infected patients on antiretroviral therapy in Southern Africa: a prospective multicohort study. J Acquir Immune Defic Syndr. 2014;67(5):547–54. doi: 10.1097/QAI.0000000000000360 25393941 PMC4231535

[9] Palich R, Makinson A, Veyri M, Guihot A, Valantin M-A, Brégigeon-Ronot S, et al. Kaposi’s Sarcoma in virally suppressed people living with HIV: an emerging condition. Cancers (Basel). 2021;13(22):5702. doi: 10.3390/cancers13225702 34830857 PMC8616070

[10] Palmieri C, Dhillon T, Thirlwell C, Newsom-Davis T, Young A-M, Nelson M, et al. Pulmonary Kaposi sarcoma in the era of highly active antiretroviral therapy. HIV Med. 2006;7(5):291–3. doi: 10.1111/j.1468-1293.2006.00378.x 16945073

[11] Imran TF, Al-Khateeb Z, Jung J, Peters S, Dever LL. Pulmonary Kaposi’s sarcoma as the initial presentation of human immunodeficiency virus infection. IDCases. 2014;1(4):78–81. doi: 10.1016/j.idcr.2014.10.002 26839780 PMC4735027

[12] Pelidis MA, Bathobakae L, Aiken A, Villegas K, Mohtadi M, Rezkalla A, et al. Pulmonary Kaposi Sarcoma in the era of antiretroviral therapy: a case series. J Med Cases. 2024 Nov;15(11):311–8. 39421229 10.14740/jmc4251 PMC11483144

[13] Freeman EE, McCann NC, Semeere A, Reddy KP, Laker-Oketta M, Byakwaga H, et al. Evaluation of four chemotherapy regimens for treatment of advanced AIDS-associated Kaposi sarcoma in Kenya: a cost-effectiveness analysis. Lancet Glob Health. 2022;10(8):e1179–88. doi: 10.1016/S2214-109X(22)00242-X 35839816 PMC9287596

[14] Adinani H, Campbell L, El-Mallawany NK, Slone J, Mehta P, Bacha J. Use of paclitaxel to successfully treat children, adolescents, and young adults with Kaposi Sarcoma in Southwestern Tanzania. Children (Basel). 2021;8(4):275. doi: 10.3390/children8040275 33918352 PMC8067189

[15] Gbabe OF, Okwundu CI, Dedicoat M, Freeman EE. Treatment of severe or progressive Kaposi’s sarcoma in HIV-infected adults. Cochrane Database Syst Rev. 2014;(9):CD003256. doi: 10.1002/14651858.CD003256.pub2 25313415

[16] Krown SE. Treatment strategies for Kaposi sarcoma in sub-Saharan Africa: challenges and opportunities. Curr Opin Oncol. 2011;23(5):463–8. doi: 10.1097/CCO.0b013e328349428d 21681092 PMC3465839

[17] Quéro L, Palich R, Valantin M-A, On Behalf Of Cancervih Working Group. The role of radiotherapy in treating Kaposi’s Sarcoma in HIV infected patients. Cancers (Basel). 2022;14(8):1915. doi: 10.3390/cancers14081915 35454820 PMC9030503

[18] Doyle M, Johnstone PA, Watkins EB. Role of radiation therapy in management of pulmonary Kaposi’s sarcoma. South Med J. 1993;86(3):285–8. doi: 10.1097/00007611-199303000-00005 7680826

[19] Lurain K, Ramaswami R, Ekwede I, Eulo V, Goyal G, Menon M, et al. Cancer immunotherapy trials network 12: pembrolizumab in HIV-associated Kaposi Sarcoma. J Clin Oncol. 2025;43(4):432–42. doi: 10.1200/JCO.24.00640 39356983 PMC11779594

[20] Ramaswami R, Polizzotto MN, Lurain K, Wyvill KM, Widell A, George J, et al. Safety, activity, and long-term outcomes of pomalidomide in the treatment of kaposi sarcoma among individuals with or without HIV infection. Clin Cancer Res. 2022;28(5):840–50. doi: 10.1158/1078-0432.CCR-21-3364 34862247 PMC8898289

[21] Tidwell J, Van Antwerp S, Bihag ZA. A race against time: rapidly progressing pulmonary Kaposi Sarcoma. Cureus. 2023;15(6):e40019. doi: 10.7759/cureus.40019 37425599 PMC10323295

[22] Ramos AL, Granado J, Calderón AI, André S, Nogueira F. Pulmonary Kaposi’s sarcoma-an atypical clinical presentation. Int J Infect Dis. 2022;115:185–8. doi: 10.1016/j.ijid.2021.11.031 34843958

[23] Sidhom SS, Laconi LA, LaFond CA, Weindorf SC. Diagnostic challenges in HHV-8-associated multicentric castleman disease in a patient with prior Kaposi Sarcoma. Dermatopathology (Basel). 2025;12(4):33. doi: 10.3390/dermatopathology12040033 41133721 PMC12550987

[24] Ramaswami R, Lurain K, Polizzotto MN, Ekwede I, Waldon K, Steinberg SM, et al. Characteristics and outcomes of KSHV-associated multicentric Castleman disease with or without other KSHV diseases. Blood Adv. 2021;5(6):1660–70. doi: 10.1182/bloodadvances.2020004058 33720337 PMC7993110

[25] Volkow P, Cesarman-Maus G, Garciadiego-Fossas P, Rojas-Marin E, Cornejo-Juárez P. Clinical characteristics, predictors of immune reconstitution inflammatory syndrome and long-term prognosis in patients with Kaposi sarcoma. AIDS Res Ther. 2017;14(1):30. doi: 10.1186/s12981-017-0156-9 28558783 PMC5450046

[26] Guan C, Shi Y, Liu J, Yang Y, Zhang Q, Lu Z, et al. Pulmonary involvement in acquired immunodeficiency syndrome-associated Kaposi’s sarcoma: a descriptive analysis of thin-section manifestations in 29 patients. Quant Imaging Med Surg. 2021;11(2):714–24. doi: 10.21037/qims-20-284 33532271 PMC7779939

[27] Restrepo CS, Martínez S, Lemos JA, Carrillo JA, Lemos DF, Ojeda P, et al. Imaging manifestations of Kaposi sarcoma. Radiographics. 2006;26(4):1169–85. doi: 10.1148/rg.264055129 16844940

[28] Gasparetto TD, Marchiori E, Lourenço S, Zanetti G, Vianna AD, Santos AASMD, et al. Pulmonary involvement in Kaposi sarcoma: correlation between imaging and pathology. Orphanet J Rare Dis. 2009;4:18. doi: 10.1186/1750-1172-4-18 19602252 PMC2720383

[29] Busi Rizzi E, Schininà V, Mazzuoli G, Armignacco O, Cecconi L. Diagnostic imaging in AIDS-related pulmonary Kaposi’s sarcoma. Radiol Med. 1998;96(4):313–7. 9972209

[30] Ibrahim Khalil A, Franceschi S, de Martel C, Bray F, Clifford GM. Burden of Kaposi sarcoma according to HIV status: A systematic review and global analysis. Int J Cancer. 2022;150(12):1948–57. doi: 10.1002/ijc.33951 35085400

[31] Nwabudike SM, Hemmings S, Paul Y, Habtegebriel Y, Polk O, Mehari A. Pulmonary Kaposi Sarcoma: an uncommon cause of respiratory failure in the era of highly active antiretroviral therapy-case report and review of the literature. Case Rep Infect Dis. 2016;2016:9354136. doi: 10.1155/2016/9354136 27872774 PMC5107221

[32] Saberian C, Lurain K, Hill LK, Marshall V, Castro EMC, Labo N, et al. Kaposi sarcoma herpesvirus viral load in bronchoalveolar lavage as a diagnostic marker for pulmonary Kaposi sarcoma. AIDS. 2024;38(8):1172–80. doi: 10.1097/QAD.0000000000003897 38564482 PMC11141217

[33] Yadav H, Shah D, Sayed S, Horton S, Schroeder LF. Availability of essential diagnostics in ten low-income and middle-income countries: results from national health facility surveys. Lancet Glob Health. 2021;9(11):e1553–60. doi: 10.1016/S2214-109X(21)00442-3 34626546 PMC8526361

[34] Ussiri EV, Lema LEK, Mbembati NAA, Mchembe M. Kaposi’s sarcoma of the lung: a case report. East Cent African J Surg. 2004;9(2):49–53.

[35] Yoo H, Lee SH, Arru CD, Doda Khera R, Singh R, Siebert S, et al. AI-based improvement in lung cancer detection on chest radiographs: results of a multi-reader study in NLST dataset. Eur Radiol. 2021;31(12):9664–74. doi: 10.1007/s00330-021-08074-7 34089072

[36] Goff CB, Dasanu CA. Changing therapeutic landscape in advanced Kaposi sarcoma: current state and future directions. J Oncol Pharm Pract. 2023;29(4):917–26. doi: 10.1177/10781552221148417 36718515

[37] Ramaswami R, Lurain K, Yarchoan R. Oncologic treatment of HIV-associated Kaposi Sarcoma 40 years on. J Clin Oncol. 2022;40(3):294–306. doi: 10.1200/JCO.21.02040 34890242 PMC8769148

[38] Dedicoat M, Vaithilingum M, Newton R. Treatment of Kaposi’s sarcoma in HIV-1 infected individuals with emphasis on resource poor settings. Cochrane Database Syst Rev. 2003;(3):CD003256. doi: 10.1002/14651858.CD003256 12917957

