# Supplementary material for: Overall survival and associated factors among patients with pulmonary Kaposi’s sarcoma in sub-Saharan Africa
Source: PLOS Glob Public Health. 2026 Jun 26;6(6):e0006741. doi: 10.1371/journal.pgph.0006741 (PMC13309032; doi:10.1371/journal.pgph.0006741)
Supplement: S1 Table — (DOCX) [file pgph.0006741.s003.docx]

|  | | | |
| --- | --- | --- | --- |
| **Covariate** | **Chi-square** | **df** | **P-value** |
| **Age** | 1.29 | 1 | 0.26 |
| **PKS** | 0.29 | 1 | 0.59 |
| **KS lesion distribution** | 0.47 | 1 | 0.49 |
| **KS lesions morphotype** | 2.00 | 1 | 0.16 |
| **Global** | 3.88 | 4 | 0.42 |
|  | | | |
